# Association of Mitochondrial DNA Polymorphisms With Pediatric-Onset Cyclic Vomiting Syndrome

**DOI:** 10.3389/fped.2022.876436

**Published:** 2022-05-24

**Authors:** Kirana Veenin, Duangrurdee Wattanasirichaigoon, Bhoom Suktitipat, Saisuda Noojarern, Patcharee Lertrit, Thipwimol Tim-Aroon, Supannee Kaewsutthi, Suporn Treepongkaruna

**Affiliations:** ^1^Division of Gastroenterology, Department of Pediatrics, Faculty of Medicine Ramathibodi Hospital, Mahidol University, Bangkok, Thailand; ^2^Division of Medical Genetics, Department of Pediatrics, Faculty of Medicine Ramathibodi Hospital, Mahidol University, Bangkok, Thailand; ^3^Department of Biochemistry, Faculty of Medicine, Siriraj Hospital, Mahidol University, Bangkok, Thailand

**Keywords:** cyclic vomiting syndrome, DNA polymorphisms, Mt16519T, Mt3010A, mitochondrial next-generation sequencing, pediatric-onset cyclic vomiting syndrome

## Abstract

**Background:**

Cyclic vomiting syndrome (CVS) is a functional gastrointestinal disorder characterized by recurrent stereotypic episodes of vomiting. The pathophysiology of CVS remains obscure. Previous studies have supported the hypotheses of mitochondrial dysfunction. However, data on association studies between mitochondrial DNA (mtDNA) polymorphisms and pediatric-onset CVS are limited and inconsistent. The aims of this study were to describe clinical characteristics, evaluate association of mtDNA polymorphisms 16519T and 3010A with pediatric-onset CVS and identify new mtDNA candidate variants.

**Methods:**

This study involved Thai patients diagnosed with CVS according to the Rome III or IV criteria before the age of 15 years. Patients' demographic data, clinical characteristics, previous investigations and treatment outcomes were obtained. Blood samples were collected for next-generation (whole exome) sequencing, followed by analysis of chromosome M (mitochondrial. Variants were filtered according to clinical significance using ClinVar and MITOMAP. mtDNA polymorphisms in 148 normal Thai individuals were used as controls.

**Results:**

Forty-eight children were enrolled in the clinical study, and 30 participated in the genetic analysis. The median age at onset and median age at diagnosis was 3.0 (1.5–5.6) and 6.3 (3.0–8.6) years, respectively. Maternal history of migraine was positive in 16.7%. About 45.7% (21 of 46) of the patients achieved complete clinical remission, with the mean symptom duration of 5.9 ± 3.3 years. The prevalence of mtDNA variants 16519T and 3010A among the patient group and Thai general population (control) were as follows: 40.0% (12/30) vs. 27.7% (*P* = 0.18) and 6.7% (2/30) vs. 0.7% (*P* = 0.07), respectively. Five known pathogenic variants were identified in 6 patients, including mtDNA 8528C in one patient who also had infantile hypertrophic cardiomyopathy. Six likely pathogenic variants were found but without statistical significance. We identified 11 variants with significant prevalence in the patient group. Though, these variants were classified as variants of unknown significance (VUS), several of them were located in mt functional regions and therefore they deserve further investigations as new candidates for association with pediatric CVS.

**Conclusion:**

There were no associations of mtDNA polymorphisms 16519T and 3010A with CVS in our pediatric cohort. Five pathogenic variants and 11 VUS were found associated with pediatric-onset CVS.

## Introduction

Cyclic vomiting syndrome (CVS) is a functional gastrointestinal disorder that affects both adults and children of all ages and presents with characteristic manifestations of acute, stereotyped and recurrent episodes of intense nausea and vomiting lasting from hours to a few days (1, 2). The worldwide prevalence of pediatric CVS is 1.9–2.3% (2), with the incidence of 3.1 per 100,000 per year (3), and the female:male ratio is 1.5:1 (1). Coexisting neuromuscular disease and developmental delay are found in up to 25% of patients with CVS, known as CVS plus (4).

CVS is strongly associated with migraine as ~25–35% of children with CVS develop migraine headache later in adulthood and there is a high prevalence of migraine headache in their families (5–9). Currently, according to the International Classification of Headache Disorders, CVS is classified as an “episodic syndrome that may be associated with migraine” (10). The etiopathogenesis of CVS remains obscure. Many underlying potential mechanisms have been postulated including mitochondrial dysfunction causing cellular energy deficit, hyperactivity of hypothalamic-pituitary-adrenal axis in response to stimulation, autonomic abnormalities, and calcium channel abnormalities (2, 6, 11, 12). Many studies supporting the hypothesis of mitochondrial dysfunction include frequent presence of metabolic abnormalities patterns of mitochondrial disorders during the episodes (6, 8), a maternal inheritance pattern (6, 9, 13, 14), and response to mitochondrial targeted therapies such as coenzyme Q10, L-carnitine and riboflavin, as well as antimigraine medications (1, 12). Boles et al. (15) reported that most cases of CVS plus had body fluid metabolites and muscle biopsies consistent with mitochondrial disorders and were strongly associated with maternal inheritance pattern.

Previous studies have shown an association between mitochondrial DNA (mtDNA) polymorphisms and pediatric-onset CVS with some conflicting results (14, 16–19). A3243G mutation was reported in a family with CVS (17). Zaki et al. (19) described 16519T polymorphism in 70% (odds ratio, 6.2) and concomitant polymorphisms of 16519T and 3010A in 29% (odds ratio, 17) of children with CVS. Subsequently, Ye et al. (18) performed mtDNA sequencing in 13 Chinese children with CVS and showed no significant differences in the prevalence of 16519T and 3010A polymorphism in children with CVS and in the control group.

The objectives of this study were to describe clinical characteristics and evaluate association of mtDNA polymorphisms 16519T and 3010A with pediatric-onset CVS and identify novel mtDNA candidate variants associated with this condition.

## Materials and Methods

This observational study was conducted at the pediatric gastroenterology clinic of the Faculty of Medicine Ramathibodi Hospital from April 2019 to April 2020. Inclusion criteria were the patients diagnosed with CVS under the age of 15 years between January 2000 and October 2019. Diagnosis of CVS was according to the Rome III (20, 21) during 2006–2015 and the Rome IV (22, 23) during 2016–2019. Patients diagnosed with CVS before 2006 were retrospectively classified according to the Rome IV. Blood samples were collected from the eligible patients for genetic analysis.

### Clinical Data Collection

The medical records of the patients were reviewed retrospectively. Demographic data, comorbidities, clinical characteristics, family history of migraine headache, hospitalizations, precipitating factors, previous investigations, treatment received, and the course of CVS were obtained. To determine the CVS outcome, the patients were interviewed in the clinic, during the study period, using the structured questionnaire that included symptoms, episodes of attack and prophylactic medications in the previous year as well as an occurrence of migraine headache. Patients who had been missing for more than 6 months were contacted by phone to collect the data using the same questions. As for patients who were lost to follow-up and could not be reached by phone, their symptoms at the last clinic visit were employed as the outcome.

CVS outcomes were classified into three groups according to the frequency of episodes during the previous year of follow-up: complete, partial, and no remission. Complete remission was defined as no episodes, partial remission as ≥50% reduction in the frequency of episodes, and no remission as <50% reduction, when compared to the symptoms at presentation.

Diagnosis of CVS plus requires at least two hard manifestations of the following: skeletal myopathy, cranial nerve abnormality, ataxia, seizure disorder, cardiomyopathy, intellectual disability, attention-deficit/hyperactivity disorder, microcephaly, and autism (4).

### Mitochondrial Genome Sequencing and Analysis

DNA was extracted from peripheral blood leukocytes using a DNA purification kit (Gentra Puregene Blood Kit; Qiagen, Hilden, Germany. Next-generation sequencing (NGS) was performed on a NovaSeq 6000 Sequencer (Illumina, San Diego, CA, USA) using SureSelect Human All Exon V5 kits (Agilent Technologies, Santa Clara, CA, USA) for target enrichment with 120-base-pair paired-end reads. The data were analyzed on Terra, a cloud-native platform for biomedical researchers. The processing steps were combined using the Workflow Description Language. Human genome reference version 38 (GRCh38) was used for alignment. The average depth of coverage was 42 with at least 30× coverage in 90% of the genome. The GATK Best Practice Workflow (version 4.1.3.0) for data processing and variant discovery was used to identify germline single-nucleotide polymorphisms (SNPs) and indels in mitochondria (chromosome M). To determine the effect of these variants, Variant Effect Predictor (ensembl-vep version 4.2) was used to annotate the variants discovered. Variants that did not pass quality control or had a heteroplasmy level of <10% (likely low clinical correlation) were eliminated from further analysis. Variants were filtered and prioritized for their clinical significance into pathogenic, likely pathogenic, variants of unknown significance, and benign. As for previously reported variants, we labeled its pathogenicity classification following its verdict as shown on the ClinVar/CLIN_SIG (https://www.ncbi.nlm.nih.gov/clinvar/docs/clinsig/) and the MITOMAP database (www.mitomap.org). We categorized the novel variants identified in the present study as VUS, and those with significant allele frequency were deposited into ClinVar database and subsequently assigned accession numbers (Supplementary Table S6). The known pathogenic variant was verified by Sanger sequencing. Primer sequences were provided in Supplementary Table S1.

### Allele Frequencies in Thai General Population

Allele frequencies of mtDNA polymorphisms were determined in 148 normal Thai controls as previously published and were used as ethnic-comparable frequencies (24). The samples were selected to represent the entire Thai population. Whole mitochondria were Sanger sequenced using 16 overlapping primer pairs.

### Statistical Analysis

Statistical analysis was performed under closed supervision and reviewed by a biomedical statistician from Department of Clinical Epidemiology and Biostatistics Faculty of Medicine Ramathibodi Hospital, Mahidol University. Data from patients performed genetic test was compared with those without genetic test. Allele frequencies between patients with CVS and the general Thai population were compared and analyzed. SPSS version 21 for Windows (IBM Corp., Armonk, NY, USA) was used for statistical analysis. Continuous variables were presented as mean ± standard deviation or median (interquartile range) and were analyzed using Mann–Whitney test. Categorical variables were presented as percentage and were analyzed by a Chi-square test. A *P*-value of <0.05 was considered statistically significant.

## Results

### Clinical Characteristics and Outcomes

Fifty-one patients were diagnosed with CVS; however, three had missing medical records. Therefore, 48 patients (54.2% female) were enrolled, and 30 participated in the genetic analysis. Exclusion from the genetic study was due to loss of contact (*n* = 14) and declining a genetic test (*n* = 4). The mean age ± SD at enrollment was 11.0 ± 4.8 (range 2.6–23) years, and the median (IQR) age at onset and at diagnosis was 3.0 (1.5–5.6) and 6.3 (3.0–8.6) years, respectively. A maternal family history of migraine headache was reported in 8 (16.7%) children. None had a family history of CVS or consanguinity (being descended from the same ancestor). There were no significant differences in age at onset, clinical remission of CVS, or maternal history of migraine between the patients who did and did not undergo genetic studies (Table 1; Supplementary Table S2).

**Table 1:** Demographic data and clinical characteristics of the patients.

**Variables**	**Pediatric-onset CVS patients**			***P*-value**
	**Total (*n* = 48)**	**With genetic study (*n* = 30)**	**Without genetic study (*n* = 18)**	
Age at enrollment, mean ± SD (y)	11 ± 4.8	11.2 ± 5.2	10.9 ± 4.3	0.65
**Gender**
Male	22 (45.8%)	10 (33.3%)	12 (66.7%)	
Female	26 (54.2%)	20 (66.7%)	6 (33.3%)	
Female: male ratio	1.2:1	2:1	0.5:1	0.025^a^
Age at onset (y), median (IQR)	3 (1.5, 5.6)	2.7 (1.5, 5.6)	3.5 (1.5, 6.3)	0.7
Age at diagnosis (y), median (IQR)	6.3 (3, 8.6)	6 (3, 8)	7.5 (2.7, 11.4)	0.16
Age of CVS resolution, mean ± SD (y)	9.6 ± 3 (*n* = 21)	9.9 ± 3.3 (*n* = 13)	9.2 ± 2.7 (*n* = 8)	0.66
Duration of follow up (y), median (IQR)	4.2 (1.1, 7.7)	5.1 (2, 8)	3.4 (0.7, 5.7)	0.06
Duration of symptom, mean ± SD (y)	5.9 ± 3.3 (*n* = 21)	6.8 ± 3.7 (*n* = 13)	4.3 ± 1.7 (*n* = 8)	0.09
Maternal history of migraine	8 (16.7%)	5 (17%)	3 (16.7%)	1.0
**Co-morbidity**
GERD	7 (14.6%)	6 (20%)	1 (5.6%)	
Asthma	4 (8.3%)	3 (10%)	1 (5.6%)	
Genetic disorders	5 (10.4%)	5 (16.7%)	0	
Neuroblastoma	1 (2.1%)	1 (3.3%)	0	
Glycogen storage type I	1 (2.1%)	1 (3.3%)	0	
**Duration of CVS symptoms (d)**
1–5	27 (56.3%)	18 (60%)	9 (50%)	0.5
>5	21 (43.8%)	12 (40%)	9 (50%)	
**Interval of symptoms (wk)**
1–4	30 (63.9%)	20 (66.7%)	10 (58.8%)	0.84
5–8	10 (21.3%)	6 (20.0%)	4 (23.5%)	
9–12	7 (14.9%)	4 (13.3%)	3 (17.6%)	

a *p < 0.05 compared between the group with vs. without genetic study*.

*d, day; GERD, gastroesophageal reflux disease; wk, week; y, year*.

Female sex was predominant among the patients who underwent genetic studies (66.7%), whereas male sex was predominant among the patients who did not (66.7%) (*P* = 0.025). Most (62.5%) patients had identifiable precipitating factors, among which psychological stress was the most common (50.0%). During vomiting episodes, the patients experienced lethargy (60.4%), abdominal pain (47.9%), hematemesis (37.5%), pallor (29.2%), hypertension (25%), headache (18.8%), and photosensitivity (4.2%). All patients diagnosed prior to establishing of the Rome IV exhibited a typical stereotyped pattern of episodes, which was included in the Rome IV but not in the Rome III diagnostic criteria.

Investigations to exclude possible organic causes were performed in all children with (1) age at onset <2 years; (2) clinical and blood chemistries suggestive of metabolic or other diseases; (3) treatment-refractory CVS. Upper GI studies were performed in 42 patients, with 4 (9.5%) reported non-specific findings (delay passage barium from 2nd to 3rd part of duodenum, pylorospasm, and gastroesophageal reflux). Abdominal ultrasonography was performed in 14 patients, with normal results. Esophagogastroduodenoscopy (EGD) was done in 33 patients with 16 (48.5%) reported abnormal but non-specific findings, including mild gastritis, mild esophagitis and prolapsed gastropathy. Twenty patients underwent brain CT scan which showed unremarkable results (Supplementary Table S2). Abnormal blood test results during the episodes included elevated blood lactate (6/29, 20.7%), hyperammonemia (4/35, 11.4%), abnormal plasma amino acid profiles (7/16, 43.8%), and abnormal urine organic acids (2/16, 12.5%) (Supplementary Table S2). Interestingly, one patient (Patient 7) developed a one-time episode of plasma citrullinemia without documented hyperammonemia but with a negative genetic analysis for urea cycle disorders and citrin deficiency, as performed by Sanger and whole-exome sequencing analysis. Treatment with a low-protein diet, ammonia-lowering agent, and CVS prophylaxis therapy alleviated this patient's symptoms. The patient was clinically well after discontinuation of treatment. Two patients had mild excretion of 3-methylglutaconic acid and lactic acid with abnormal urinary organic acid profile, and were treated as CVS with complete remission at 9 and 7 years of age, respectively. Genetic studies performed in all suspected cases did not reveal a specific metabolic disorder.

Prophylactic therapy included amitriptyline (22/45), cyproheptadine (14/45) and propranolol (12/45). At the median duration of follow-up of 4.2 years (IQR 1.1, 7.7 years), 21 out of 46 patients achieved complete clinical remission; the mean age at clinical remission was 9.6 ± 3.0 years, and the mean symptom duration was 5.9 ± 3.3 years. Migraine headache developed after CVS remission in 3/21 (14%) patients. Partial remission and no remission occurred in 21 and 4 patients, respectively. Among all patients, 13 still required hospitalization for episodic attacks.

### CVS Plus

Six patients (83% female) were diagnosed with CVS plus, with a median age at onset of 4.3 (1.7–7.3) years; this age did not differ from that of the other patients. Two patients with CVS plus showed lactic acidosis. Three (50%) patients had a maternal history of migraine headache compared with 11.9% (5/42) of the non-CVS plus patients (*P* = 0.05; Supplementary Table S2). Complete remission rate was lower in CVS plus (1/6 or 16.7% vs. 20/40 or 50%, *P* = 0.19); and the frequency of no remission was higher in CVS plus (1/6 or 16.7% vs. 3/40 or 7.5%, *P* = 0.44).

### Mitochondrial DNA Polymorphisms

The prevalence of mtDNA variants 16519T and 3010A among the patient group and Thai general population (control) were as follows: 40.0% (12/30) vs. 27.7% (*P* = 0.18) and 6.7% (2/30) vs. 0.7% (*P* = 0.07), respectively (Supplementary Table S3).

Mitochondrial genome analysis revealed 5 known pathogenic variants in 6 patients, 6 likely pathogenic variants in 30 patients (Tables 2, 3), and 312 VUS (with ≥10% level of heteroplasmy).

**Table 2:** Five pathogenic mtDNA variants identified and clinical findings of the patients.

**Case; ID**	**Age (y); Sex**	**mtDNA variants**	**Gene and description**	**Nucleotide change**	**Amino acid change**	**Reported morbidity**	**Clinical findings^a^**	**Remission** **of CVS**
1; 3	15; M	12811	NADH dehydrogenase subunit 5	T-C homoplasmy	p.Tyr159His	Possible LHON factor	Cornelia de Lange syndrome; onset of CVS at 2 y; no visual problem	Partial
2; 50	3; F	12811	NADH dehydrogenase subunit 5	T-C homoplasmy	p.Tyr159His	Possible LHON factor	Onset of CVS at 11 m	Partial
3; 47	9; F	8528	ATP synthase F0 subunit 8/6	T-C heteroplasmy	p.Trp55Arg (ATPs8) p.Met1Thr (ATPs6)	Infantile hypertrophic cardiomyopathy	Thrombocytopenia at 6 m; HCM at 10 m (EF 72%); recurrent vomiting at 3 y; acute encephalopathy, elevated CSF lactic acid level at 8.5 y; periodic hyperlactatemia; GDD, ADHD, and light colored and fine hair	No
4; 26	13; F	5814	tRNA cysteine	T-C homoplasmy	Non-coding	Encephalopathy	Onset of CVS at 5 y; normal psychomotor development without history of alteration of consciousness	Complete; at age 12 y
5; 7	14; F	3394	NADH dehydrogenase subunit 1	T-C homoplasmy	p.Tyr30His	Associated with LHON	Onset of CVS at 2 y; normal psychomotor development	Complete; at age 10 y
6; 5	13; F	827	12S ribosomal RNA	A-G homoplasmy	Non-coding	Associated with deafness	Onset of CVS at 3 y; normal psychomotor development; normal hearing	Complete; at age 4 y

a *None of the patients had lactic acidosis and/or family history of LHON, with the exception of case 3 (patient ID 47)*.

*AD, Alzheimer's disease; ADHD, attention deficit and hyperactivity disorder; EF, ejection fraction; GDD, global developmental delay; HCM, hypertrophic cardiomyopathy; LHON, Leber hereditary optic neuropathy; m, months; MELAS, mitochondrial encephalomyopathy, lactic acidosis, and stroke-like episodes; PD, Parkinson's disease; y, years*.

**Table 3:** Six likely pathogenic mtDNA variants and their frequency identified in this study.

**mtDNA variants**	**Description**	**Nucleotide change**	**Amino acid change**	**Known morbidity**	**CVS (%)**	**Control (%)**	***p*-value**
14783	Cytochrome b	T-C	p.Leu13Leu	NA	16 (53.3)	50.7	0.75
15043	Cytochrome b	G-A	p.Gly99Gly	Major depressive disorder -associated	16 (53.3)	51.4	0.84
15301	Cytochrome b	G-A	p.Leu185Leu	Found in tissue tumor (somatic)	16 (53.3)	52.7	0.69
12372	NADH dehydrogenase subunit 5	G-A	p.Leu12Leu	Altered brain pH/sporadic Creutzfeldt–Jakob disease	2 (6.7)	9.5	0.74
15670	Cytochrome b	T-C	p.His308His	Found in breast tumor tissue (somatic)	1 (3.3)	0.7	0.31
15346	Cytochrome b	G-A	p.Leu200Leu	NA	1 (3.3)	4.1	1

*NA, not available*.

Among those with pathogenic variants, one patient (Patient 47) with T8528C (97% heteroplasmy) had clinical presentations that matched the reported morbidity. The patient presented with infantile-onset skin bruising owing to thrombocytopenia (20,000–60,000/mm^3^), hypertrophic cardiomyopathy (HCM) with outflow tract obstruction, and developmental delay. The HCM was gradually improved over time. At the age of 6 years, symptoms of cyclic vomiting began and platelet storage pool defect was suspected as evidenced by abnormal platelet aggregation and normal number of platelets. At the age of 8.5 years, the patient developed acute loss of consciousness with preceding 1-day history of agitation and confusion. Investigations revealed normal brain magnetic resonance imaging and magnetic resonance spectroscopy (MRI and MRS); elevated lactate level in cerebrospinal fluid (2.7 mmol/L) whilst normal blood lactate (1.2 mmol/L); elevated serum creatine kinase (1,079 U/L); normal profiles of blood ammonia, plasma amino acids, acylcarnitine profile and urinary organic acids; and mild HCM with ejection fraction of 76%. The patient recovered to her baseline within a couple days of general supportive care. Both parents had a normal echocardiogram. The mother harboring the T8528C variant at a low level of heteroplasmy (Supplementary Figure S1) reported no essential morbidities.

Among the 312 VUS, 11 were found to be significantly more prevalent among the CVS group than control group and had not been previously reported in patients with CVS (Table 4; Supplementary Tables S4, S5). These included 8 variants located at hypervariable (HV) segment of the control region and a variant of 12s ribosomal RNA gene, a non-coding nucleotide (Table 4; Supplementary Table S4).

**Table 4:** Eleven variants of unknown significance (VUS) with important allele frequency.

**mtDNA variants**	**Locus**	**Description**	**Nucleotide change**	**Amino acid change**	**CVS (*n* = 30)**	**Control (*n* = 148)**	***p*-value**
235	MT-HV2	Hypervariable segment 2	A-G	Non-coding CR	3 (10%)	2 (1.4%)	0.034^a^
247	MT-HV2	Hypervariable segment 2	GA-G	Non-coding CR	4 (13.3%)	0	0.001^a^
513	MT-HV3	Hypervariable segment 3	GCA-G	Non-coding CR	11 (36.7%)	0	0
1736	MT-RNR2	16S ribosomal RNA	A-G	Non-coding 16S rRNA	3 (10%)	0	0.004^a^
8270	MT-NC7	Non-coding nucleotides	CACCCCCTCT-C	Non-coding	5 (16.7%)	0	0
14668	MT-ND6	NADH dehydrogenase subunit 6	C-T	p.Met2Met	2 (6.7%)	0	0.028^a^
16159	MT-HV1	Hypervariable segment 1	C-A	Non-coding CR	2 (6.7%)	0	0.028^a^
16179	MT-HV1	Hypervariable segment 1	CAA-C	Non-coding CR	2 (6.7%)	0	0.028^a^
16230	MT-HV1	Hypervariable segment 1	A-G	Non-coding CR	2 (6.7%)	0	0.028^a^
16274	MT-HV1	Hypervariable segment 1	G-A	Non-coding CR	4 (13.3%)	4 (2.7%)	0.029^a^
16319	MT-HV1	Hypervariable segment 1	G-A	Non-coding CR	4 (13.3%)	4 (2.7%)	0.029^a^

a *p < 0.05 when compared between CVS and control groups*.

*CR, control region; HV, hypervariable segment*.

Among the 6 CVS plus patients, 1 pathogenic (T8528C), as mentioned above), 3 likely pathogenic and 3 VUS were found but there was no statistically significant difference in term of prevalence when compared with non-CVS plus patients (Supplementary Table S5).

## Discussion

Clinical and genetic studies on pediatric CVS among Asian affected population are limited and underrepresented in the literature. The existing genetic data are also conflicting which may reflect the scarcity of information. To our knowledge this is the largest cohort of Thai pediatric CVS and contribute as one of the milestone studies from Asian affected population.

In this cohort, we demonstrated similar clinical features to those in previous reports including vomiting characteristics and long duration of symptoms before diagnosis (5, 18, 25). However, the mean age of onset was slightly lower as compared to previous pediatric CVS studies (8, 18, 25). Maternal migraine headache was found in 16.7% in this study. Family history of migraine headache in pediatric CVS ranged from 11.9% in Chinese study (18) to 66–72% in western studies (8, 26). During the episodes, abnormal laboratory findings included elevated blood lactate, hyperammonemia, abnormal plasma amino acid profiles and urine organic acids.

Almost half of the patients achieved complete clinical remission at the mean age of 9.6 years, and among this group, the mean clinical course of CVS was 5.9 years. This outcome is consistent with our previous report (27) and other reports (25, 26). Three patients (14%) developed migraine after CVS remission while other studies reported around 25–35% (6, 7). Only one of the three patients underwent a mtDNA genetic test, which showed no 16519T or 3010A polymorphism.

It should be noted that CVS plus is not generally employed in daily clinical practice; however, in the present study of etiopathogenesis of CVS, it could raise concerns relating to differential diagnosis for the affected individuals, in particular pediatric population. Therefore, in this study, we decided to analyze this subgroup and search for potential distinctive etiology, if there was any. We found a higher prevalence of maternal migraine among the CVS plus group compared to non-CVS plus group (50 vs. 11.9%, *P* = 0.05), suggesting a higher rate of maternal inheritance in patients with CVS plus than in non-CVS plus. Patients with CVS plus had a lower complete remission rate (16.7 vs. 50%, *P* = 0.19); and a higher frequency of no remission (16.7 vs. 7.5%, *P* = 0.44). There were no statistical differences of the outcomes probably due to the small number of the patients in CVS plus group. These data suggest that pathogenesis of CVS plus may differ from non-CVS plus and may deserve more extensive investigations for etiologic diagnosis, including exome and mitochondrial genome sequencing for causal-disease relationship.

It is postulated that underlying mtDNA polymorphism might impact energy metabolism both in the resting state and a hyperexcitability state, including stress circumstance (6, 12). The mitochondrial genome encompasses 24 rRNA and tRNA genes and 13 genes encoding proteins involved in oxidative phosphorylation (complex I–V) (28). Some mitochondrial variants are judged as pathogenic or as having a “causality” effect, while others may contribute to the risk of developing multifactorial disorders or are considered to have an “association” effect.

Our study showed no significant association between mtDNA 16519T or 3010A SNPs and pediatric CVS. This result supports the finding in Chinese children with CVS (18) and contrasts the findings in Caucasian reports (16, 19). Explanations for these discrepancies include the small sample sizes, unintentional bias of subject recruitment, and differences in allele frequencies of the SNPs of interest among different ethnicities. More studies in Asian CVS children are required to confirm our findings.

The known pathogenic variants identified in this study (Table 2) have been reported in association with several conditions including Leber hereditary optic neuropathy; deafness; myopathy; cardiomyopathy; and encephalopathy, but not CVS. We have provided the first description of the association of these variants with pediatric-onset CVS. There were two (6.7%) occurrences of mtDNA T12811C variants in the CVS group compared with 4.73% in the control group, suggesting a strong correlation between this variant and CVS compared with other pathogenic SNPs.

Including our patient (Patient 47), seven patients with the mtT8528C allele have been identified (29, 30). All patients had infantile onset HCM, and about half (4/7 patients) had rapid progression leading to infantile death or requiring heart transplantation at a young age. To our knowledge, the present patient was the oldest living individual affected with mtT8528C-associated HCM. This study is the first to show an association between the mtT8528C allele and CVS, platelet dysfunction, abnormal hair, and behavioral disorders. Mitochondrial disorders caused by nuclear gene mutations are more severe than those caused by mtDNA mutations (31), with the exception of some rare variants including mtT8528C (29, 30). Given there has no previous association study of mtT8528C and CVS before, it suggests that mtT8528C and other mt pathogenic variant(s) can also has CVS as one of its related manifestations and that mt pathogenic variant(s) should be considered as possible SNPs associated with pediatric CVS regardless of the presence/absence of classical manifestation of mt disorders.

We found that patients with CVS tended to have at least one pathogenic variant when compared with the general population. Although the allele frequency of each pathogenic variant was not different from those in the control group, we cannot rule out a possible correlation between these variants and CVS/other manifestations. When the data of all SNPs were combined, the aggregate prevalence of pathogenic SNPs in the CVS group was 20% (6/30), which was higher than that in the reference population (9.5%) (*P* = 0.1). The absence of an association between the likely pathogenic variants and CVS is a straightforward finding because neither the individual nor aggregate prevalence of the variants differed between the two groups. Our data indicate that NGS with a mitochondrial analysis pipeline could be powerful for genomic research in CVS and related disorders.

Of the 11 VUS, one missense variant in complex I might affect mitochondrial function to some extent. Among the eight alleles found in HV segments of the control region, five were in the highly polymorphic HV1. However, part of the HV1, mt16040–16188 containing very low sequence variability, is important in mtDNA replication and possibly translation. A specific segment, mt16157–16172, is an important functional region: the termination-associated sequence (14). Therefore, the mtC16159A and mtCAA16179C variants (Table 4) might interfere with mitochondrial replication and translation. Among the variants in HV3, GCA513G of the D-loop (control region) appears to be a hotspot for somatic and germline mtDNA mutation and may reflect ancient mutation or ancestry (MITOMAP, https://www.mitomap.org/); therefore, it may or may not possess functional effects. We observed that C14668T variant was present in two patients with CVS plus and this variant was residing in the functional locus, MT-ND6 (Table 4; Supplementary Table S4). This may suggest that the variant deserves high priority for association study of mt polymorphisms with pediatric-onset CVS. Further studies are required to elucidate the functional consequences of this allele.

The strength of our study included whole mt genome being analyzed and having ethnic-comparable mt genome reference, which is important factor to avoid bias and increase reliability of the analysis for association study. However, we acknowledge some limitations. First, the clinical data were obtained by medical chart review and were incomplete because of the nature of this retrospective study. Second, although this is one of the few largest cohorts of pediatric CVS with whole mitochondrial genome analysis, the sample size was small. Third, statistical significance does not guarantee clinical relevance. Fourth, functional investigation was not performed to elaborate the pathological effects of the VUS identified. Fifthly, the presence of some individuals with CVS in the control group cannot be totally excluded because the individuals might not be aware of CVS during their childhood and the symptoms could be misinterpreted as infection related. Finally, the studied specimens were obtained from peripheral blood, which may not represent the mitochondrial genotypes in other tissues.

Because pediatric CVS is a rare condition, we urge multicenter national and/or regional networks to perform a larger cohort study with an ethnic-comparable reference population, which may lead to a stronger association study. Additionally, development of a simple and less sophisticated method of mitochondrial functional analysis is needed for research in the fields of CVS and mitochondrial disorders.

## Conclusion

In conclusion, the mtDNA polymorphisms 16519T and 3010A were not associated with pediatric-onset CVS in this pediatric cohort. However, 5 known pathogenic variants and 11 novel VUS were shown to be associated with pediatric-onset CVS. Our data suggest that NGS with a mitochondrial analysis pipeline is a powerful tool for mtDNA polymorphism study in CVS research.

## Data Availability Statement

The data presented in the study are deposited in the ClinVar Database, accession numbers are shown in the Supplementary Table S6.

## Ethics Statement

The studies involving human participants were reviewed and approved by Ramathibodi Hospital Human Research Ethics Committee (MURA2019/310). Written informed consent to participate in this study was provided by the participants' legal guardian/next of kin.

## Author Contributions

KV collected and analyzed the clinical data, performed the bioinformatics analysis of the patient group, and prepared the manuscript draft. BS supervised the bioinformatics analysis. PL supervised and SK performed the bioinformatics analysis of the control population. SN performed the bioinformatics analysis and sequencing. TT-A assisted with the data analysis. ST obtained a research grant. ST and DW designed the overall concept of the study, supervised the data analysis, and critically revised the manuscript. All authors reviewed and approved the final manuscript.

## Funding

This study was funded by the Faculty of Medicine Ramathibodi Hospital, Mahidol University (RF_62086).

## Conflict of Interest

The authors declare that the research was conducted in the absence of any commercial or financial relationships that could be construed as a potential conflict of interest.

## Publisher's Note

All claims expressed in this article are solely those of the authors and do not necessarily represent those of their affiliated organizations, or those of the publisher, the editors and the reviewers. Any product that may be evaluated in this article, or claim that may be made by its manufacturer, is not guaranteed or endorsed by the publisher.
