# Supplementary material for: Association of Mitochondrial DNA Polymorphisms With Pediatric-Onset Cyclic Vomiting Syndrome
Source: Front Pediatr. 2022 May 24;10:876436. doi: 10.3389/fped.2022.876436 (PMC9170898; doi:10.3389/fped.2022.876436)
Supplement: Supplementary file 1 [file Data_Sheet_1.pdf]

Supplementary Material: Methods-Results

Association of mitochondrial DNA polymorphisms with pediatric-onset cyclic vomiting syndrome. Kirana Veenin et al.

Supplementary Method (Table S1) and Results (Table S2-S5 and Figure S1)

Table S1. Primer sequence for Sanger sequencing of the mitochondrial variant identified in the present study

Primer name	Forward (5'-3')	Reverse (5'-3')	Mt region covered	Product (bp) [a]
**m.700-m.910**	TAGACGGGCTCACATCACCC	AGTTTTAGCTTTATTGGGGAGGG	RNR1	361
**m.830-m1070**	GAACAAGCATCAAGCACGCA	AGCTGTGGCTCGTAGTGTTC	RNR1	409
**m.3150-m.3780**	GTTCAACGATTAAAGTCCTACGTGA	CAAGGTCGAAGGGGGTTCG	RNR2, RNR3, TER, TL1, NC1, ND1	868
**m.3790-m.4540**	AAACTCAAACTACGCCCTGAT	GGCAGCTTCTGTGGAACGA	ND1, TI, TQ, TM, ND2	993
**m.5530-m.6160**	CATCGCCCTTACCACGCTAC	TATAGCAGATGCGAGCAGGAGTA	TW, TA, TN, OLR, TC, TY, NC5, CO1	799
**m.8280-m.8580**	TGGAGCAAACCACAGTTTCATGC	TGTTGGGTGGTGATTAGTCGG	NC7, TK, NC8, ATP8, ATP6	482
**m.8650-m.9250**	CACAATCCTAGGCCTACCCG	GGCCTAGTATGAGGAGCGTTA	ATP6	784
**m.10990-m.11130**	ACCTATTTAGCTGTTCCCCAACC	GGAAGTATGTGCCTGCGTTC	ND4	306
**m.12250-m.12880**	ACAGAGGCTTACGACCCCTTA	GGTGAGGCTTGGATTAGCGT	TS2, ND5, TL2	790

[a] PCR conditions: initial denaturation at 95°C for 5 min; 35 cycles of denaturation at 95°C for 30 secs, annealing at 60-61°C for 30 secs, extension at 72°C for 35 secs; and final extension at 72°C for 7 min.

ATP6, ATP synthase F0 subunit 6; ATP8, ATP synthase F0 subunit 8; CO1, Cytochrome c oxidase subunit I; NC1, non-coding nucleotides, locus 1; NC5, non-coding nucleotides, locus 5; NC7, non-coding nucleotides, locus 7; NC8, non-coding nucleotides, locus 8; ND1, NADH Dehydrogenase subunit 1; ND2, NADH dehydrogenase subunit 2; ND4, NADH dehydrogenase subunit 4; ND5, NADH dehydrogenase subunit 5; OLR, L-strand origin; RNR1, 12S ribosomal RNA; RNR2, 16S ribosomal RNA; RNR3, 5S-like sequence; TA, tRNA alanine; TC, tRNA cysteine; TER, Transcription terminator; TI, tRNA isoleucine; TK, tRNA lysine; TL1, tRNA leucine 1; TL2, tRNA leucine2; TM, tRNA methionine; TN, tRNA asparagine; TQ, tRNA glutamine; TS2, tRNA serine2; TW, tRNA tryptophan; TY, tRNA tyrosine

TABLE S2. Clinical characteristics of each individual patient (N=48)

ID	Sex	Age (years)				CVS plus	comorbidity	Mat. migraine	Lactic acidosis	Other tests
		current	onset	diagnosis	remission					
With genetic study (N=30)										
1	F	21.4	2	7	10	no	Ehlers-Danlos syndrome, GERD	no	NA	NA
2	M	18.4	2	6.5	16	no	CP, ADHD, GERD	no	yes	PAA, UOA, CT brain- normal; MELAS mutation- negative
3	M	15.2	4	5.7		no	Cornelia de Lange syndrome, GERD	no	no	PAA, UOA- normal
5	F	13	3	4.3	4.8	no	-	no	NA	NA
6	M	10.3	0.6	1.5		no	GERD, pharyngeal incoordination	no	no	PAA- low citrulline and elevated glycine and L-glutamine; UOA, CT brain- normal; OTC gene- no mutation
7	M	14.3	2	6.3	10.8	no	History of citrullinemia	no	NA	PAA- elevated glutamine and citrulline; UOA, CT brain, whole exome analysis- normal
8	F	23	2	14.7	15.5	no	-	no	no	PAA- elevated glutamine with normal citrulline and arginine; UOA- normal; OTC gene- no mutation
10	M	12	3	4	9.2	no	-	yes	no	UOA- ketone bodies and mild elevation of 3-methylglutaconic acid; PAA- elevated glutamine with normal citrulline and arginine
11	F	9.3	1	1.3	7	no	-	no	no	UOA- marked excretion of lactic acid and ketone bodies; PAA- normal
13	F	9.8	1.5	3.5	7	no	-	no	no	NA
17	F	8.6	3.7	5		no	neuroblastoma	no	no	CT brain- normal
18	F	11.2	0.5	4.3	8	no	CP due to birth asphyxia	no	no	NA

19	M	13.7	8	9.3		yes	microcephaly, CP, epilepsy, GERD	yes	yes	UOA- normal
20	F	16	10	11		no	-	no	no	NA
22	M	8.7	3.3	3.5		no	DiGeorge syndrome	no	no	CT brain- normal
23	M	12.2	8	8.3	11	no	-	no	NA	NA
24	F	4.8	1.5	1.8		no	-	no	yes	PAA- severe ketosis; UOA-marked excretion of ketone bodies, CT brain- normal
25	F	3.4	0.5	0.9		no	FTT	no	no	PAA, UOA, CT brain- normal
26	F	13.3	5.8	7.3	12	no	-	no	NA	NA, CT brain-one small tiny cyst at pineal gland
27	M	7.2	5.2	6.3		no	6q25.2q27 deletion, GDD	no	yes	PAA, UOA, CT brain- normal
36	F	18.8	2	2.2	7	yes	CP, epilepsy, GERD	yes	NA	NL; CT brain- normal
41	F	13.8	6.8	7.2	10	no	-	no	NA	NA
44	F	10.3	7	9.8		yes	GDD, epilepsy	no	NA	NA
45	F	2.6	0.7	2		yes	GDD, epilepsy, truncus arteriosus	yes	no	NA
46	M	10.8	7	8		no	-	no	NA	CT brain- normal
47	F	9	3	8.7		yes	thrombocytopenia and platelet dysfunction, ADHD, hypertrophic cardiomyopathy, GDD and acute encephalopathy	no	yes	PAA, UOA, DBS, CT brain, chromosome microarray and trio whole exome study- normal
48	F	4	2.3	3.7		no	-	no	no	CT brain- normal
49	F	10	2	8		no	GSD type 1a, GERD	no	NA	NA
50	F	3	0.9	1.3		no	-	yes	no	PAA, UOA- normal
51	F	8.4	5.5	6.5		yes	GDD, epilepsy	no	no	CT brain- normal

Without genetic study (N=18)										
4	F	15.3	10	11.3	11.6	no	-	no	NA	NA
9	M	8.4	0.08	0.7		no	-	yes	no	PAA, UOA, CT brain- normal
12	F	18	7	12.3	14	no	choledochal cyst	no	NA	PAA- elevated glutamine and glycine
14	F	13	1.5	2		no	-	no	no	CT brain- normal
15	M	7.5	1.5	2.8	5	no	primary hypothyroidism, GERD	no	no	NA
16	F	9.6	2	4.9	8	no	-	no	yes	PAA- decrease BCAA probably due to low intake; UOA- normal
21	M	10.7	2	10.3		no	-	no	no	NA
29	M	13.6	3	13.3		no	-	no	no	NA
30	M	11.3	2	8		no	-	yes	NA	NA
31	M	9	4	8.2	9	no	-	no	NA	NA
32	M	3.2	1.2	2.4		no	-	no	NA	MEALS gene- negative
33	M	8	4	6.3	7.3	no	-	no	NA	NA
34	M	14	5	6.4	9	no	-	no	NA	CT brain- normal
37	M	14.3	5	13.4		no	-	no	NA	NA
38	M	1.5	0.4	1.4		no	delayed speech	no	no	PAA, UOA, CT brain- normal
39	M	10.4	6	6.9	10	no	ADHD, asthma	no	no	CT brain- normal
40	F	11.7	8	11.7		no	-	no	no	NA
42	F	16	8	10.3		no	growth hormone deficiency	yes	NA	CT brain- normal

ADHD, attention deficit and hyperactivity disorder; BCAA, branched-chain amino acids; CP, cerebral palsy; DBS, dried blood spot analysis for acyl carnitine profiles; FTT, failure to thrive; GDD, global developmental delay; GSD, glycogen storage disease; GERD, gastroesophageal reflux disease; Mat. migraine, maternal history of migraine; OTC, ornithine transcarbamylase; PAA, plasma amino acids; UOA, urine organic acids; y, years

TABLE S3. List of main variants identified in this study and its description

Variants	Locus	Description	Nucleotide change	Amino acid change	Reported disease	Homoplasmy	Heteroplasmy	GB frequency	Patient ID	N
**Target variants (N=2)**										
16519T	MT-CR	entire control region (including displacement loop)	T-T	noncoding CR	CVS	(+)	(-)	36.9%	1,2,3,6,7,13, 19,24,25, 36, 46,50	12
3010A	MT-RNR2	16S ribosomal RNA	G-A	noncoding 16SrRNA	CVS	(+)	(-)	14.4%	19,36	2
**Pathogenic variants (N=5)**										
827G	MT-RNR1	12S ribosomal RNA	A-G	noncoding 12S rRNA	deafness -associated	(+)	(-)	2.5%	5	1
3394C [1]	MT-ND1	NADH dehydrogenase subunit 1	T-C	p.Tyr30His	associated with LHON	(+)	(-)	1.3%	7	1
5814C [1]	MT-TC	tRNA cysteine	T-C	noncoding tRNA	encephalopathy	(-)	(+)	0.3%	26	1
8528C	MT-ATP8/6	ATP synthase F0 subunit 8/6	T-C	Trp55Arg (ATPs8) Met1Thr (ATPs6)	Infantile hypertrophic cardiomyopathy	(+)	(+)	0.0%	47	1
12811C [1]	MT-ND5	NADH dehydrogenase subunit 5	T-C	p.Tyr159His	possible LHON factor	(+)	(-)	1.3%	3,50	2
**Likely pathogenic variants (N=6)**										
14783C	MT-CYB	Cytochrome b	T-C	p.Leu13Leu	NA	NA	NA	21.2%	2,3,6,7,10,11 ,18,19,24,25, 36,41,44,47, 50,51	16

15043A	MT-CYB	Cytochrome b	G-A	p.Gly99Gly	major depressive disorder -associated	(+)	(-)	23.6%	2,3,6,7,10,11,18,19,24,25,36,41,44,47,50,51	16
15301A	MT-CYB	Cytochrome b	G-A	p.Leu185Leu	found in tissue tumor (somatic)	NA	NA	28.7%	2,3,6,7,10,11,18,19,24,25,36,41,44,47,50,51	16
12372A	MT-ND5	NADH dehydrogenase subunit 5	G-A	p.Leu12Leu	altered brain pH/sCJD	(+)	(-)	13.4%	17,46	2
15670C	MT-CYB	Cytochrome b	T-C	p.His308His	found in breast tumor tissue (somatic)	NA	NA	1.7%	11	1
15346A	MT-CYB	Cytochrome b	G-A	p.Leu200Leu	NA	NA	NA	0.8%	49	1
**VUS (N=11)**										
235G	MT-HV2	HV2	A-G	noncoding CR	found in prostate tumor tissue (somatic)	NA	NA	3.0%	1,13,27	3
247G	MT-HV2	HV2	GA-G	noncoding CR	NA	NA	NA	NA	2,23,47,48	4
513G	MT-HV3	HV3	GCA-G	noncoding CR	NA	NA	NA	NA	6,8,10,13,22,23,27,41,44,47,48	11
1736G	MT-RNR2	16S ribosomal RNA	A-G	noncoding 16SrRNA	NA	NA	NA	2.8%	1,13,27	3
8270C	MT-NC7	non-coding nucleotides	CACCCCCTCT-C	noncoding	NA	NA	NA	NA	2,5,22,26,49	5
14668T	MT-ND6	NADH dehydrogenase subunit 6	C-T	p.Met2Met	depressive disorder associated	(+)	(-)	4.1%	19,36	2
16159A	MT-HV1	HV1	C-A	noncoding CR	NA	NA	NA	0.0%	20,49	2
16179C	MT-HV1	HV1	CAA-C	noncoding CR	NA	NA	NA	NA	20,49	2
16230G	MT-HV1	HV1	A-G	noncoding CR	NA	NA	NA	3.3%	22,26	2

16274A	MT-HV1	HV1	G-A	noncoding CR	found in prostate tumor tissue (somatic)	(-)	(+)	2.4%	6,11,27,49	4
16319A	MT-HV1	HV1	G-A	noncoding CR	NA	NA	NA	5.9%	1,11,13,27	4

[1] pathogenicity with recently conflicting result

AD, Alzheimer disease; ADHD, attention deficit and hyperactivity disorder; CR, control region; GB, Gen Bank; HV, hypervariable segment; LHON, Leber hereditary optic neuropathy; lactic acidosis and stroke-like episodes; PD, Parkinson disease; sCJD, Sporadic Creutzfeldt-Jakob disease; T2DM, diabetes mellitus type 2

TABLE S4. mt variants of unknown significance (VUS) in each individual patient (n=30)

ID	mtDNA variants
1	73G, 152C, 235G, 263G, 1736G, 2706G, 6641C, 7028T, 8860G, 11719A, 12705T, 14364A, 16092C, 16223T, 16290T, 16319A, 16362C
2	42TC, 73G, 247G, 489C, 2706G, 3552A, 4715G, 5821A, 6338G, 7028T, 7196A, 7853A, 8270C, 8584A, 8701G, 8860G, 9540C, 10400T, 10873C, 11719A, 11914A, 12705T, 13263G, 14318C, 16223T, 16298C, 16327T
3	73G, 150T, 199C, 489C, 2831T, 4048A, 4071T, 4164G, 5351G, 5460A, 6455T, 6680C, 7028T, 7419A, 7684C, 7853A, 8701G, 8860G, 9540C, 9824C, 10400T, 10873C, 11719A, 12405T, 12705T, 15773A, 16192T, 16223T, 16297C
5	73G, 499A, 2706G, 4820A, 7028T, 8270C, 8343G, 8860G, 9707C, 11719A, 12398T, 13590A, 14587G, 15535T, 16051G, 16136C, 16183C, 16189C, 16217C, 16218T
6	73G, 143A, 285CAAA, 489C, 513G, 930C, 942G, 2706G, 2833G, 4086T, 7028T, 8152A, 8701G, 8860G, 9214G, 9540C, 9685C, 10400T, 10873C, 11719A, 12705T, 12973T, 13651G, 13914A, 15853T, 16209C, 16223T, 16233G, 16274A, 16304C
7	73G, 150T, 153G, 385G, 2706G, 4491A, 7028T, 8155A, 8701G, 8860G, 9540C, 10400T, 11719A, 12237T, 12705T, 14308C, 16095T, 16148T, 16223T, 16234T, 16355T, 16356C, 16362C
8	73G, 513G, 2706G, 3421A, 3970T, 4086T, 6392C, 6962A, 7028T, 7747T, 8149G, 8860G, 9053A, 9548A, 10310A, 10609C, 11215T, 11515T, 11719A, 12136C, 12406A, 12882T, 13759A, 13928C, 16108T, 16129A, 16162G, 16172C, 16304C
10	73G, 146C, 489C, 513G, 2706G, 3912G, 4071T, 4850T, 5378G, 6455T, 7028T, 8701G, 8860G, 9449T, 9540C, 9824C, 10400T, 11665T, 11719A, 11932T, 12091C, 12705T, 14053G, 16172C, 16223T, 16291T, 16311C
11	73G, 146C, 234G, 447G, 489C, 1780C, 2706G, 5426C, 5774C, 7028T, 7762A, 7961C, 8502G, 8701G, 8860G, 9142A, 9540C, 10400T, 11083G, 11719A, 12705T, 16093C, 16223T, 16274A, 16319A
13	73G, 152C, 207A, 235G, 513G, 1719A, 1736G, 2706G, 7028T, 8459G, 8860G, 11719A, 11974G, 12705T, 13788T, 14067T, 16086C, 16223T, 16290T, 16319A, 16362C
17	73G, 150T, 2706G, 4856C, 5231A, 5417A, 7028T, 8860G, 11719A, 12358G, 12705T, 13742T, 14178C, 15080G, 16111T, 16223T, 16257A, 16292T, 16294T
18	73G, 489C, 1664A, 2706G, 5108C, 5492C, 7028T, 7598A, 7939T, 8508G, 8701G, 8860G, 9055A, 9116C, 9540C, 10873C, 11447A, 11482C, 11719A, 12705T, 12940A, 14552G, 16093C, 16223T, 16258C, 16290T, 16311C, 16390A
19	73G, 152C, 489C, 2706G, 3206T, 4883T, 5178A, 5582G, 6975C, 7028T, 7609C, 8473C, 8701G, 8859T, 8860G, 9540C, 10400T, 10873C, 11719A, 12705T, 14668T, 14979C, 16129A, 16223T, 16362C

20	73G, 185A, 189G, 709A, 2706G, 5836G, 7028T, 8271ACC, 8277C, 8279C, 8860G, 10031C, 11061T, 11719A, 12950G, 13269G, 13681G, 13879C, 14322G, 14766T, 16179C,CA, 16183C,ACCC,ACCCC, 16189C, 16311C, 16390A, 16399G
22	73G, 153G, 210G, 513G, 709A, 2706G, 3537G, 4385G, 6960T, 7028T, 8270C, 8584A, 8860G, 9950C, 11719A, 13145A, 13395G, 14364A, 16140C, 16183C, 16189C, 16209C, 16230G, 16266A
23	73G, 248delA, 514-515delCA, 2389T, 2706G, 3398C, 3970T, 6392C, 6962A, 7028T, 8860G, 10310A, 10609C, 11719A, 12406A, 12882T, 13576G, 13928C, 15247T, 16189C, 16234T, 16304C
24	73G, 150T, 199C, 4164G, 5351G, 5460G, 6455T, 7028T, 7684C, 9540C, 9824C, 10400T, 10792G, 10873C, 11087C, 11719A, 12405T, 12705T, 16129A, 16192T, 16223T, 16297C
25	73G, 143A, 146C, 151T, 489C, 1607C, 2706G, 4853A, 7028T, 8440G, 8701G, 8718G, 8838A, 8860G, 9540C, 9615C, 10400T, 10873C, 11719A, 12705T, 13759A, 14605G, 16129A, 16140C, 16203G, 16223T, 16271C
26	73G, 2706G, 7028T, 8270C, 8860G, 9123A, 11719A, 12732C, 14518G, 16217C, 16261T
27	73G, 152C, 235G, 513G, 1736G, 2706G, 7028T, 8860G, 11719A, 12705T, 13759A, 16223T, 16274A, 16290T, 16319A, 16362C, 16527T
36	73G, 489C, 2706G, 4883T, 5178A, 7028T, 8701G, 9540C, 10400T, 10873C, 11719A, 12705T, 14668T, 16223T, 16249C, 16311C, 16362C
41	73G, 146C, 150T, 210G, 489C, 504C, 514-515delCA, 2706G, 4646C, 5108C, 6581G, 7028T, 8701G, 8860G, 9053A, 9058G, 9210G, 9449T, 9540C, 10373A, 10400T, 10873C, 11482C, 11719A, 12612G, 12705T, 15940delC, 16108T, 16129A, 16223T
44	73G, 150T, 279C, 489C, 513G, 862G, 930C, 2706G, 4318A, 4769G, 7028T, 8701G, 8860G, 9254G, 9540C, 10324C, 10400T, 10873C, 11016A, 11719A, 11908G, 12705T, 12711G, 12804C, 12973T, 13973T, 15530C, 15802G, 16093C, 16129A, 16209C, 16223T, 16224C, 16278T, 16325C, 16362C
45	73G, 195C, 2706G, 4491A, 6770G, 7028T, 8545A, 8860G, 10166C, 10609C, 10877T, 11719A, 13145A, 13287T, 13359A, 14790G, 15885T, 16249C, 16259T, 16288C, 16301T, 16304C, 16390A
46	73G, 150T, 385G, 2706G, 5231A, 5263T, 5417A, 7028T, 8772C, 8860G, 11719A, 12358G, 12705T, 12771A, 16183C, 16189C, 16223T, 16257A
47	73G, 152C, 225A, 248delA, 489C, 514-515delCA, 2706G, 3200C, 3714G, 4385T, 4772C, 7028T, 7433T, 8701G, 8853G, 8860G, 9127G, 9380A, 9512T, 9540C, 10274C, 10400T, 10679G, 10873C, 11719A, 11914A, 12354C, 12705T, 14110C, 14974T, 15691G, 16086C, 16129A, 16209C, 16223T, 16272G
48	73G, 248delA, 514-515delCA, 2706G, 3970T, 4715G, 6392C, 6515C, 6962A, 7028T, 8860G, 9053A, 10310A, 10609C, 11719A, 12406A, 12771A, 12882T, 13759A, 13928C, 14750G, 16129A, 16171G, 16172C, 16304C
49	73G, 146C, 150T, 709A, 2706G, 3497T, 3571T, 7028T, 8270-delACCCCCTCT, 8772C, 8860G, 11719A, 12882T, 14053G, 14659T, 16092C, 16140C , 16180_16181delAA, 16189C, 16217C, 16274A, 16311C, 16335G

50	73G, 150T, 199C, 489C, 2706G, 4048A, 4071T, 4164G, 5351G, 5460G, 6228T, 6455T, 6680C, 7028T, 7684C, 7853A, 8701G, 8860G, 9540C, 9824C, 10232G, 10400T, 10873C, 11719A, 12405T, 12705T, 16129A, 16192T, 16223T, 16297C
51	73G, 489C, 1342T, 2706G, 5812G, 6253C, 7028T, 7759C, 8269A, 8701G, 8860G, 9540C, 10873C, 11719A, 11810T, 12705T, 12732C, 13020C, 13468T, 14793G, 16223T, 16304C, 16344T, 16362C, 16381C

ID, patient ID

TABLE S5. Summary of pathogenic and likely pathogenic variants and VUS identified in each patient

Case	ID	Pathogenic variants	Likely pathogenic variants	VUS	CVS plus (yes, no)
1	1	-	-	235G; 1736G; 16319A	no
2	2	-	14783C; 15043A; 15301A	247G; 8270C	no
3	3	12811C	14783C; 15043A; 15301A	-	no
4	5	827G	-	8270C	no
5	6	-	14783C; 15043A; 15301A	513G; 16274A	no
6	7	3394C	14783C; 15043A; 15301A	-	no
7	8	-	-	513G	no
8	10	-	14783C; 15043A; 15301A	513G	no
9	11	-	14783C; 15043A; 15301A; 15670C	16274A; 6319A	no
10	13	-	-	235G; 513G; 1736G; 16319A	no
11	17	-	12372A	-	no
12	18	-	14783C; 15043A; 15301A	-	no
13	19	-	14783C; 15043A; 15301A	14668T	yes
14	20	-	-	16159A; 16179C	no
15	22	-	-	513G; 8270C; 16230G	no
16	23	-	-	247G; 513G	no
17	24	-	14783C; 15043A; 15301A	-	no
18	25	-	14783C; 15043A; 15301A	-	no
19	26	5814C	-	8270C; 16230G	no
20	27	-	-	235G; 513G; 1736G; 16274A; 16319A	no
21	36	-	14783C; 15043A; 15301A	14668T	yes
22	41	-	14783C; 15043A; 15301A	513G	no
23	44	-	14783C; 15043A; 15301A	513G	yes
24	45	-	-	-	yes
25	46	-	12372A	-	no
26	47	8528C	14783C; 15043A; 15301A	247G; 513G	yes
27	48	-	-	247G; 513G	no
28	49	-	15346A	8270C; 16159A; 16179C; 16274A	no
29	50	12811C	14783C; 15043A; 15301A	-	no
30	51	-	14783C; 15043A; 15301A	-	yes

TABLE S6. List of. ClinVar accession number of the pathogenic, likely pathogenic and VUS variants identified in the present study

Variants	Reported or Novel	ClinVar accession[a]	dbSNP
m.12811T>C	reported	VCV000065510.5	rs199974018
m.8528T>C	reported	VCV000009640.2	rs387906422
m.5814T>C	reported	VCV000030001.4	rs200077222
m.3394T>C	reported	VCV000009725.4	rs41460449
m.827A>G	reported	VCV000009634.6	rs28358569
m.14783T>C	reported	VCV000140588.1	rs193302982
m.15043G>A	reported	VCV000140589.1	rs193302985
m.15301G>A	reported	VCV000140591.1	rs193302991
m.12372G>A	reported	VCV000522717.2	rs2853499
m.15670T>C	reported	VCV000143927.1	rs193302997
m.15346G>A	reported	VCV000143883.1	rs527236180
m.1027A>G	reported	VCV000178943.1	rs727504555
m.235A>G	novel (present study)	SCV002104251	NA
m.247GA-G	novel (present study)	SCV002104256	NA
m.513GCA-G	novel (present study)	SCV002106315	NA
m.1736A>G	novel (present study)	SCV002106316	NA
m.8270delACCCCCTCT	novel (present study)	SCV002107184	NA
m.14668C>T	novel (present study)	SCV002106317	NA
m.16159C>A	novel (present study)	SCV002107182	NA
m.16179delAA	novel (present study)	SCV002107183	NA
m.16230A>G	novel (present study)	SCV002107185	NA
m.16274G>A	novel (present study)	SCV002107186	NA
m.16319G>A	novel (present study)	SCV002107187	NA

[a] the novel variants were deposited into ClinVar database and accession numbers were given (https://www.ncbi.nlm.nih.gov/clinvar/)

Supplementary Result: FIGURE S1. Sequences of six pathogenic variants identified in the seven patients

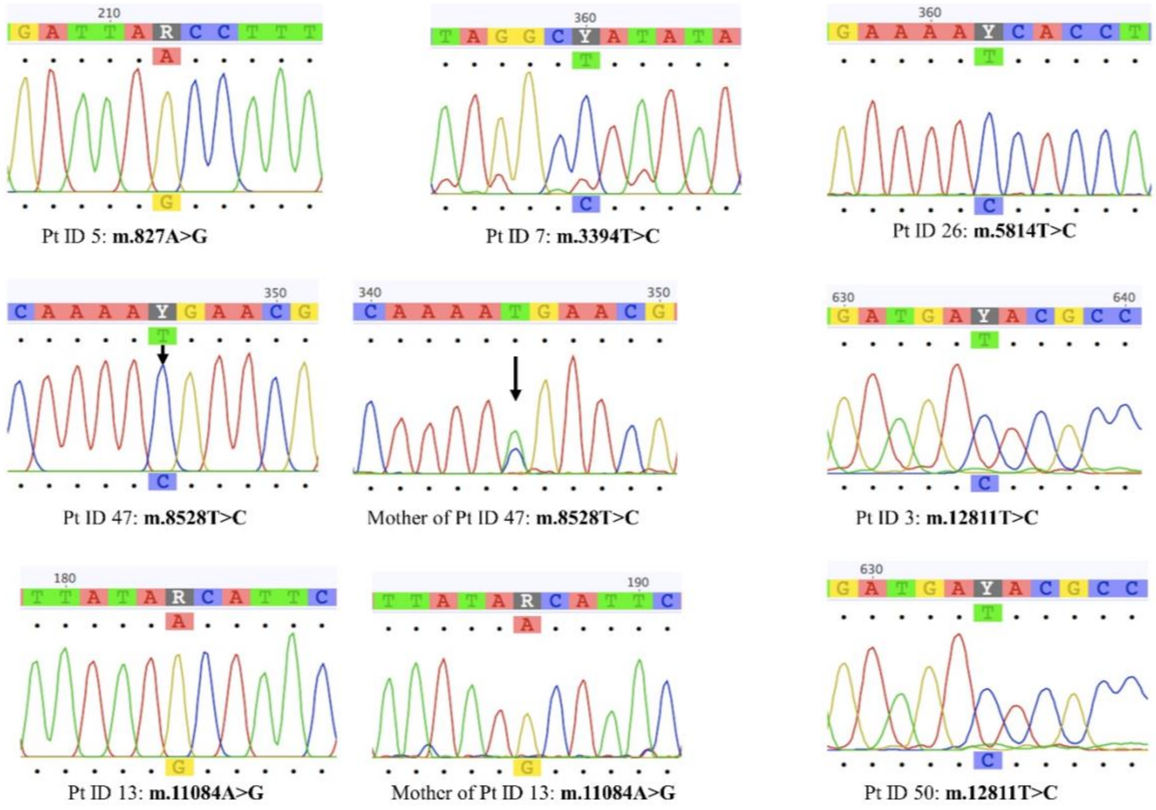

Each figure piece is self-explanatory. Noted in Patient ID47, only pathogenic allele mtT8528C was demonstrated by Sanger sequencing, whereas this variant was detected with high level of heteroplasmy (at 97%) by whole exome sequencing. The asymptomatic mother of patient ID47 was however shown to have lower degree of heteroplasmy. The mtA11084G in patient ID13 was confirmed inherited from the patient's healthy mother. All the PCR sequencing were performed using DNA extract from peripheral blood.
